# Inhibitor of Differentiation-3 and Estrogenic Endocrine Disruptors: Implications for Susceptibility to Obesity and Metabolic Disorders

**DOI:** 10.1155/2018/6821601

**Published:** 2018-01-08

**Authors:** Mayur Doke, Vincent Avecilla, Quentin Felty

**Affiliations:** Department of Environmental & Occupational Health, Florida International University, Miami, FL, USA

## Abstract

The rising global incidence of obesity cannot be fully explained within the context of traditional risk factors such as an unhealthy diet, physical inactivity, aging, or genetics. Adipose tissue is an endocrine as well as a metabolic organ that may be susceptible to disruption by environmental estrogenic chemicals. Since some of the endocrine disruptors are lipophilic chemicals with long half-lives, they tend to bioaccumulate in the adipose tissue of exposed populations. Elevated exposure to these chemicals may predispose susceptible individuals to weight gain by increasing the number and size of fat cells. Genetic studies have demonstrated that the transcriptional regulator inhibitor of differentiation-3 (ID3) promotes high fat diet-induced obesity in vivo. We have shown previously that PCB153 and natural estrogen 17*β*-estradiol increase ID3 expression. Based on our findings, we postulate that ID3 is a molecular target of estrogenic endocrine disruptors (EEDs) in the adipose tissue and a better understanding of this relationship may help to explain how EEDs can lead to the transcriptional programming of deviant fat cells. This review will discuss the current understanding of ID3 in excess fat accumulation and the potential for EEDs to influence susceptibility to obesity or metabolic disorders via ID3 signaling.

## 1. Introduction

Obesity is considered to be one of the most significant public health challenges of the 21st century [1]. Population based studies have shown the association between obesity and metabolic disorders which include diabetes, insulin resistance, coronary heart disease, and fatty liver disease [2–5]. Obesity is a medical condition defined as the excess accumulation of body fat. The World Health Organization (WHO) stated in a previous report that approximately 650 million people were obese, 18 years and older in the world during 2016 [6]. The economic impact of obesity on health care costs has been estimated to be more than 200 billion dollars in the US [7]. The European Union (EU) has expected the associated costs of obesity and diabetes to be over 18 billion euros per year from exposure to endocrine disrupting chemicals [8]. Currently, there is an unmet need to understand how endocrine disrupting chemicals contribute to susceptibility to obesity and metabolic disorders.

Estrogen is a class of hormones with a myriad of functions including the regulation of adipose tissue and metabolism [9]. Adipose tissue is a complex metabolic, endocrine organ. The relative contribution of adipose tissue to steroid production is significant with adipose tissue producing up to 100% of circulating estrogen in postmenopausal women and 50% circulating testosterone in premenopausal women [10, 11]. Aromatase enzyme is expressed in preadipocytes and adipose tissue stromal cells. Aromatase catalyzes the conversion of androgens (androstenedione and testosterone) to estrogen (estrone and estradiol) in the endoplasmic reticulum [12–15]. Both estrogen receptor (ER) subtypes ER*α* and ER*β* are found in adipose cells [16, 17]. 17*β*-Estradiol (E2) signaling occurs through both genomic (nuclear) and nongenomic (extra-nuclear) pathways [18, 19]. Nuclear estrogen receptors consist of ER*α* and ER*β*, while membrane estrogen receptors (mERs: GPER, GPR30, ER-X, and Gq-mER) are mostly G protein-coupled receptors [20–26]. Since the adipose tissue is an endocrine organ, it may be susceptible to EEDs. Endocrine disruptors are chemicals that alter hormone production or function including phytoestrogens, heavy metals, and anthropogenic chemicals. More specifically, EEDs include compounds such as genistein, arsenic, DES, PCBs, phthalates, and bisphenol A (BPA). Relative binding affinities of ortho, para-DDE, hydroxylated PCB, BPA, and DES have been shown to be significantly weaker than E2 in binding to nuclear ERs and mERs like GPR30 [27–31]. Moreover, EEDs have been shown to mimic estrogenic activity and interfere with the endocrine system through these receptor signaling pathways [32–34]. A recent study of offspring from pregnant women exposed to DES showed an association with obesity [35]. Early-life exposure to DES was shown to increase obesity in mice at 4–6 months of age compared to control mice [36]. Thus, elevated exposure to EEDs is of concern because they may predispose susceptible individuals to weight gain by increasing the number and size of fat cells. This review is focused on linking the obesogenic effects of EEDs to ID3 signaling leading to increased fat accumulation or obesity.

## 2. Transcription Regulator ID3

The molecular factors that contribute to the development of excess body fat in response to endocrine disruption have yet to be fully elucidated. Genetic studies have demonstrated that the transcriptional regulator ID3 promotes high fat diet-induced obesity* in vivo*. The ID (inhibitor of differentiation) family of small proteins consists of four genes (ID1–ID4). ID1 and ID3 have been shown to regulate cell growth, self-renewal, senescence, angiogenesis, and neurogenesis [37–42]. Depending on the cellular context, ID1 and ID3 have been shown to exhibit overlapping functions as dual gene knockout combinations have demonstrated redundancy [43]. ID1 and ID3 have been shown to be coexpressed in early development of the cell cycle progression, angiogenesis, and neurogenesis in the mouse model [40–42]. Amongst PCB congeners, PCB153 has been found to be one of the largest contributors to total PCB body burden in humans and a diet-dependent obesogen in the experimental model [44, 45]. We have previously demonstrated that PCB153 modulates ID3 expression and phosphorylation [46]. ID3 is highly expressed in the embryonic tissue and highly proliferating and undifferentiated adult cells [43]. We and others have shown that PCB153 increases oxidative stress or reactive oxygen species (ROS) that mediate ID3 expression [47, 48]. Exposure to estrogenic chemicals has been shown to increase ROS in the nucleus in which they modify the surrounding DNA necessary for transcriptional activation of cell growth genes [49–51]. In other words, ROS that we have already shown to be induced by treatment with PCB153 may be involved in ID3 mediated transcription regulation. EEDs have been shown to increase ROS production in adipocytes. Di-(2-ethyl hexyl)phthalate (DEHP) increased ROS in rat adipocytes [52]. The plastic chemical BPA which has been linked to obesity in both human and animals studies was demonstrated to increase ROS levels in mesenchymal stem cells involved in the process of adipogenesis [53, 54]. Mitochondria are a major source of ROS production in mammalian cells [55]. Although other endogenous ROS sources besides mitochondria such as NADPH oxidase exist, we have shown that estrogenic chemicals increase mitochondrial ROS [56]. Furthermore, the presence of ER*α* and ER*β* in mitochondria may potentially be targets of EEDs contributing to oxidative stress [57–59]. Although there is evidence linking EEDs exposure to increased mitochondrial (mt) ROS, it is unclear whether it is responsible for redox-sensitive phosphorylation of ID3 upon exposure to PCB153.

**Figure 1:**
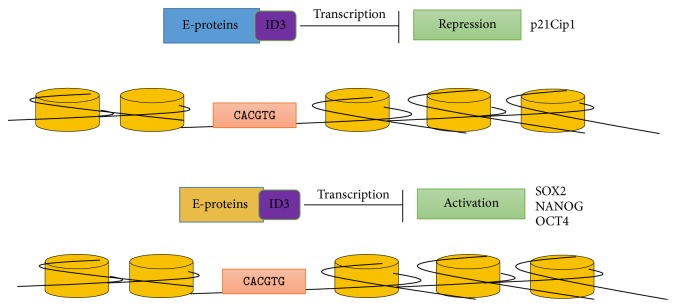
ID3 regulates a variety of cellular processes which includes cellular growth, senescence, apoptosis, differentiation, angiogenesis, and neoplastic transformation. This figure illustrates the ID3 interaction with E-proteins. The ID3 protein controls transcription of genes like p21^Cip1^, OCT4, SOX2, and NANOG by binding to the E-proteins and preventing them from interacting with the E-box sequence on the DNA.

Transcriptional regulation by ID3 ultimately functions to increase cell proliferation and preserve multipotency. ID3 mediated gene regulation governing these processes in adipocytes and stem/progenitor fat cells provides a possible explanation for how ID3 promotes high fat diet-induced obesity in the experimental model [60]. ID3 protein-protein interactions occur via the helix loop helix (HLH) motif. ID3 protein interactions block the DNA binding activity of basic HLH (bHLH) transcription factors encoded by the genes TCF3, TCF4, and TCF12. TCF3 gene encodes for E12, E47 proteins. TCF4 gene encodes for E2-2, and TCF12 gene encodes for HEB proteins in humans [61]. E12, E47, E2-2, and HEB proteins are a class I bHLH proteins which consists of basic DNA binding domain. These E-proteins specifically recognize and bind to Ephrussi-box (E-box) sequences (CANNTG) on the DNA [62, 63]. ID3 has been most often reported to interact with proteins encoded by TCF3 gene [39]. ID3 protein-protein interactions can regulate transcription by E-proteins preventing their binding and subsequent activation of target gene promoters [39]. ID3 has frequently been described throughout the literature as an inhibitor of gene expression. For example, ID3 promotes cells to pass through cell cycle checkpoints by inhibiting the expression of cell cycle inhibitor gene p21^Cip1^ (Figure 1) [64]. However, ID3 can also act as a positive transcriptional regulator depending on the cellular context. E-proteins suppress the expression of embryonic genes OCT4, SOX2, and NANOG leading to cell differentiation [65]. As shown in Figure 1, ID3 can increase the expression of these embryonic genes by repressing TCF3. We have demonstrated that ectopic overexpression of ID3 increased OCT4 and SOX2 expression and resulted in a cell population positive for molecular stem cell markers CD133^+^ VEGFR3^+^ CD34^+^ [66]. Based on these findings, ID3 can maintain undifferentiated cells by increasing the expression of embryonic pluripotency factors via repression of TCF3. Since ID3 is a transcription regulator of genes involved in both cell proliferation and stemness, EEDs may facilitate the uncontrolled proliferation of adipocytes through ID3 contributing to obesity or metabolic disorders.

Exposure to EEDs may also exert their negative health effects by altering epigenetic marks including DNA methylation and histone acetylation ultimately influencing gene expression in adipose tissue cells. Epigenetic transgenerational inheritance of obesity has been demonstrated in animals exposed to EEDs: BPA and phthalates [67]. Early-life exposures to EEDs like DES and PCB153 are known to alter DNA methyltransferase activity [68]. Since altered DNA methylation have been found in PCB153 exposed adipocytes [69], it is biologically plausible that chromatin modifications including acetylation/deacetylation of histones are another way for ID3 to regulate transcription. ID proteins have been shown to promote acetylation and transcriptional activity by recruiting histone acetyltransferase (HATs): (i) CREB-binding protein (CBP), (ii) p300 (E1A binding protein p300), and/or P300/CBP-associated factor (PCAF) to the chromatin [70]. Moreover, ID proteins interact with another chromatin modifying the protein, ZRF1 [71]. These evidences suggest that ID3 may regulate transcription through interactions with both transcription factors and chromatin modifying proteins. Although a molecular risk factor for obesity from exposure to EEDs is not known, we propose that ID3's demonstrated involvement in HFD-induced obesity coupled with its functional role in transcription regulation of cell proliferation and stemness makes it a likely candidate for environmental disruption by EEDs.

## 3. Role of ID3 in Adipose Tissue

**Figure 2:**
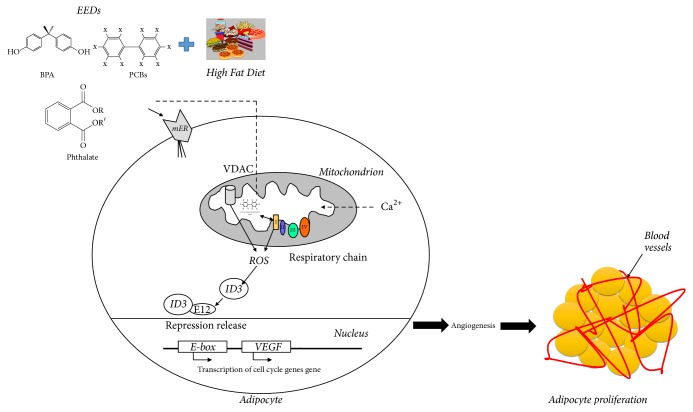
ID3 regulated adipocyte proliferation. EEDs are known to increase mitochondrial reactive oxygen species production. Since ID3 is redox-sensitive protein, ROS increases ID3 expression. ID3 binds to E12 protein and releases the repression of transcription of downstream genes like VEGF which may induce angiogenesis. This may share a similar pathway with HFD-induced obesity model in which ID3 increases obesity based on angiogenesis that support adipocyte proliferation. The scheme shows ID3 transcription regulation of genes involved genes in HFD-obesity.

ID3 is expressed during embryonic development but declines throughout the maturation of the embryo [41]. Multipotency of adipocyte progenitor cells has been shown to be maintained by ectopic expression of ID3 [72]. Two types of adipose tissue in the body consist of white adipose tissue (WAT) and brown adipose tissue (BAT) [73]. The storage of excess energy in the form of triglycerides occurs in WAT. Exposure to a HFD or excessive energy intake can increase total body WAT by the accumulation of triglycerides that may lead to obesity through chronic exposure [74]. Several studies indicate a novel role for ID3 as a regulator of obesity. HFD-induced obesity was shown to be reduced in ID3−/− knockout (ID3 KO) compared to wild-type mice [75]. Exposure to a HFD showed increased ID3 expression in the expanded visceral WAT of only wild-type mice. Hence, ID3 KO prevented the observed increase in obesity from exposure to the HFD. ID3 KO mice had a significant reduction in VEGFA protein. The decrease of VEGFA was attributed to repression of its gene promoter due to the loss of ID3 in knockout mice. This study concluded that the loss of ID3 prevented HFD-induced obesity via inhibiting VEGFA expression and adipose tissue angiogenesis necessary to support the expansion of visceral fat. Moreover, recently researchers have demonstrated in a mouse model that ID1 protein, which is also a member of ID protein family, suppresses peroxisome proliferator-activated receptor g coactivator 1a (PGC1a) which controls BAT-mediated thermogenesis and stimulates energy storage in adipocytes. Eventually, ID1 promotes obesity [76]. Our investigations of PCB153 exposed endothelial cells demonstrated that redox-sensitive ID3 signaling contributes neovascularization and vascular sphere formation [46]. Based on these evidences, we propose that the increase in fat cells from exposure to EEDs depends on ID3 mediated increase in blood vessels needed for the growth of fat tissue (Figure 2) [77].

Besides supporting an increase in blood supply to the fat tissue, ID3 may directly impact transcriptional programming of fat stem cells exposed to EEDs. For instance, the HFD-induced proliferation of fat stem cells was shown to be significantly inhibited in Id3 KO mice [60]. Since ID3 is important in the maintenance of multipotency, environmental disruption from exposure to EEDs may increase self-renewal of fat stem cells. The abundance of fat stem cells coupled with ID3 mediated proliferation may in turn lead to fat accumulation. Genetic knockout of cyclin-dependent kinase inhibitor p21^Cip1^ produces adipocyte hyperplasia and obesity in mice [78]. Consistent with this study, HFD significantly increased ID3 expression and decreased p21^Cip1^ mRNA in adipocyte progenitors [60]. Hence, Id3 KO mice may be protected from HFD-induced obesity due to the absence of adipocyte progenitor cells and/or high levels of p21^Cip1^. Together these evidences implicate ID3 as a molecular risk factor of obesity susceptible to environmental disruption especially to EEDs that accumulate in the fat tissue.

## 4. Role of ID3 in Inflammation

The inflammatory process across multiple organ systems has been implicated in the development of obesity and metabolic disorders. For example, insulin resistance and systemic inflammation result from complex interactions between the vasculature, adipose tissue, and immune cells [79, 80]. As described previously, ID3 is essential for vasculogenesis as well as the self-renewal of fat cells. However, its role in inflammation brings additional complexity to its role in disease pathogenesis. Inflammatory factors produced during obesity are a major pathway for developing metabolic complications. The induction of cytokines has been observed in population studies of obesity and/or metabolic syndrome (MetS). Several lines of evidence suggest that ID3 mediated inflammation may contribute to obesity through an imbalance in pro- and anti-inflammatory factors secreted by fat cells. Dysregulated expressions of adipokines have been observed in Id3 KO mice [81]. Adiponectin is a known adipokine that is one of the proinflammatory factors secreted by adipocytes and implicated in the development of obesity and metabolic disorders as described in the following studies. Low circulating levels of adiponectin have been linked to several components of the metabolic syndrome like intra-abdominal body fat distribution, hyperlipidemia, low high-density lipoprotein (HDL) levels, and insulin resistance/type 2 diabetes [81]. Adiponectin gene expression [82] and circulating adiponectin levels [83] are lower in patients with type 2 diabetes than in nondiabetic individuals. Population studies have shown that circulating adiponectin concentrations are reduced in obese individual [82–84]. In a cross-sectional study of men and women who were obese and lean, the negative relationship between plasma adiponectin and visceral fat (measured by computed tomography scan) was significantly stronger than that with subcutaneous fat [85]. Consistent with the observed association of low adiponectin in obesity and metabolic disorders, ID3 was found to suppress the transcription of adiponectin in adipocytes [81].

**Figure 3:**
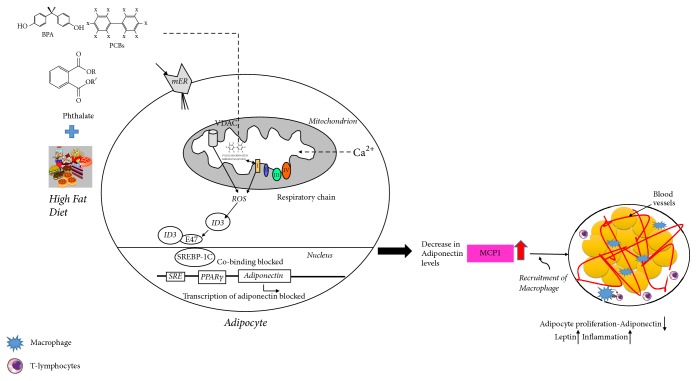
ID3 mediated inhibition of adiponectin contributes to excess adipocytes. Low circulating levels of adiponectin have been linked to several components of the metabolic syndrome. The figure illustrates how EEDs may lead to decrease in adiponectin levels via ID3 redox signaling. Elevated levels of ID3 protein bind to E47, which further prevent cobinding with SREBP-1C and may result in blocking the transcription of adiponectin gene. Additionally ID3 demonstrates regulation of MCP-1 causing increase in inflammation and adipocyte proliferation.

Interleukins are also potent mediators of the inflammatory response in immune and vascular cells. ID3 is known to regulate the production of IL-5, IL-6, IL-8, and IL-10 which have been observed in population studies of obesity and/or MetS [60, 86, 87]. Monocyte chemoattractant protein-1 (MCP-1) regulates inflammation in visceral adipose tissue and is increased in both obese mice and humans [60, 88]. Expression of MCP-1 was shown to be mediated by ID3 in fat stem cells, and MCP-1 promoted the recruitment of macrophages to the adipose tissue [60]. MCP-1 transgenic mice increased macrophage proliferation and induced expression of adipokines such as tumor necrosis factor-*α* and interleukin-6 in adipocytes [89, 90]. Furthermore, pretreatment adipocytes with adiponectin significantly reduced expression of MCP-1 when exposed to lipopolysaccharides [91]. This demonstrates the inverse relationship between adiponectin level and MCP-1 expression. Based on this evidence, dysregulation of adipose tissue inflammation via ID3 may potentially be susceptible to exposure by EEDs. Repression of adiponectin, as well as activation of MCP-1 expression by EEDs, may create an imbalance of adipose tissue inflammatory factors. Individuals exposed to EEDs may be susceptible to ID3 mediated inflammation from macrophage recruitment via MCP-1 as well as loss of anti-inflammatory adipokine adiponectin [92]. The contribution of ID3 to vasculogenesis, energy metabolism, and the immune system make it a unique molecular factor to study in obesity because it cuts across these complex organ systems. Based on these studies, we have illustrated potential mechanisms that EEDs may share with HFD-induced obesity via ID3 control of adipose inflammation shown in Figure 3.

## 5. Role of Estrogen in Obesity

Although EEDs such as DES have been shown to be detrimental to an experimental model of obesity, the meaning of these studies becomes even more complex from studies of the natural estrogen, E2. Estrogen is essential in the regulation of metabolism and regional distribution of adipose tissue [93, 94]. The presence of ER*α* and ER*β* in adipocytes established the initial link between estrogen levels and adipose cell function [95]. Population based studies have shown that ER*α* expression was reduced in adipocytes from obese compared to normal weight females [96]. Adipose cells also constitute a significant fraction of total estrogen synthesis in males and postmenopausal females [97]. It has been shown that a mutation in the aromatase gene results in estrogen deprivation and fat accumulation in men [98]. In support of the protective effect of E2 in obesity, HFD fed mice exposed to E2 showed less adipogenesis [99]. E2 treatment decreased expression of lipogenic genes such as SREBP-1c (Sterol Regulatory Element Binding Protein 1c) and LXR-*α* (Liver X Receptor *α*), a positive regulator of SREBP-1c in adipose tissue [100]. Essentially, SREBP-1c can promote the expression of lipogenic genes such as FAS and acetyl-CoA carboxylase (ACC-1) [101]. However, by lowering the expression of SREBP-1c, E2 control the adipose cells differentiation. Estrogen and metabolic hormones like leptin, insulin, and adiponectin are interlinked through hypothalamic GnRH neuronal network. ER*α* localizes along with leptin receptors in the hypothalamic region of CNS [102, 103]. Recently, it has been shown that specific silencing of ER*α* in hypothalamus results in increased food intake and a decline in energy expenditure resulting in development obesity via cholecystokinin signaling [104]. Peripheral effects are exerted directly on adipose tissue and include effects on thermogenesis and lipid synthesis through ER*α* [98]. Moreover, estrogen regulates the secretion and circulating levels of leptin in blood through local production of estrogen by adipocytes. Although the role of E2 is beneficial with respect to suppressing the accumulation of fat cells, EEDs could disrupt estrogen signaling pathways [105, 106]. Therefore, the involvement of EEDs in interfering estrogen signaling cannot be ignored.

## 6. Estrogenic Endocrine Disrupting Chemicals and Obesity

Many studies have discussed the effects of EEDs on reproductive, immune, and nervous systems, and several excellent reviews are available on these topics [107–109]. We focus here on the possible involvement of EEDs in the development of obesity and metabolic syndrome (MetS). MetS is a term for a combination of disorders that may include impaired glucose tolerance or insulin resistance, dyslipidemia, high blood pressure, and obesity [110]. Key factors are abdominal obesity and insulin resistance, where normal insulin levels are insufficient to reduce circulating levels of glucose or triglycerides.

The molecular mechanisms behind a possible involvement of EEDs, the so-called obesogens, in obesity are poorly understood. There are over one hundred chemicals, both natural and synthetic, classified as endocrine disruptors that exhibit estrogenic activity and are recognized as environmental estrogen [111–114]. The health concern over environmental estrogen is partly based on the pivotal role that natural estrogen such as E2 plays in reproduction and development. The synthetic estrogen diethylstilbestrol (DES), which was used by physicians to prevent miscarriages and in the livestock industry to enlarge poultry, cattle, and sheep [113], has become classic environmental estrogen used to model exposure in animals and humans. DES is now recognized to have led to dysfunction in reproductive organs, abnormal pregnancies, reduced fertility, immune system disorders, and depression in the daughters of women who received treatment [115]. A variety of agricultural and industrial chemicals known as organochlorines possess estrogenic activity [111, 112]. For example, o,p′-DDT an isomer of the technical grade pesticide DDT which accounts for up to 20% of the mixture [113] has been reported to be estrogenic in several species [111]. The organochlorine, polychlorinated biphenyl (PCB) which was primarily used in electrical transformers and capacitors, was reported to impair reproduction in marine mammals that fed on PCB contaminated fish [111]. Although DDT and PCBs have been banned and are not used in the United States, wildlife and humans can still be exposed due to their stability in the environment. The lipophilic property of these synthetic compounds allows them to enter the food chain as well as bioaccumulate in the adipose tissue of animals and humans. For example, a combination of environmental estrogen, such as DDT, PCBs, chlordane, and dieldrin, have been found in bald eagles [111]. In human breast milk and adipose tissue, residues of DDT, PCBs, and other organochlorine pesticides have been documented [116–119].

Evidence supporting the contribution of EEDs varies from correlative to direct induction of fat tissue. In the Faroe Islands, a study on children showed an association between prenatal dietary exposure to PCBs and DDE (a breakdown product of DDT) and increased body weight [120]. Early-life exposure to BPA was associated with increased body weight in young children [121]. Also, exposure to PCBs during fetal development or at a young age was linked with increased weight in boys and girls at puberty [122]. In support of these correlative studies,* in vivo* models showed that fetal exposure to either BPA or DES predisposes adult rodents to develop obesity [123, 124], while exposure to certain PCB congeners has also been shown to predispose animals to weight gain [125]. Moreover, Zanella et al. demonstrated exposure to genistein induced adipose tissue development in low fat diet mice and adipocyte proliferation in 3T3-L1 cell line [126]. Exposure to estrogenic PCB153 has also been shown to worsen HFD-induced obesity and nonalcoholic fatty liver disease (NAFLD) in mice [44].* In vitro* studies on adipocyte cell lines like 3T3-L1 cell line have shown that very low concentrations of BPA increase adipocyte differentiation and lipid accumulation in a dose-dependent manner [127]. Based on these studies, prenatal exposure to EEDs may reprogram the fate of the stem cell compartment responsible for adipocyte cells, which we will describe later with respect to ID3.

## 7. ID3 Mediated Obesity from Exposure to EEDs

Estrogenic hormone replacement therapy has been shown to protect against many age-related changes in adipose tissue remodeling at menopause [128]. However, fetal exposure to EEDs has been demonstrated to have an opposite effect in the rodent model, which we have described previously. These effects may be in part directed by nuclear receptor signaling. BPA is a estrogenic chemical and has binding affinity for ER*α* and ER*β* [105]. Experiments on adult mice showed that BPA acts via ER*α* causing an imbalance in the basal metabolic regulation of body in addition to increased fat mass [129]. Both ER signaling and synthesis of estrogen by aromatase cannot be ruled out as targets of disruption by EEDs. Our novel discovery shows that PCB153 alters the expression and activation of ID3 through ROS formation [46]. In addition, we demonstrated that the Pyk2-mediated estrogen-induced ID3 mRNA contributes to the growth of microvascular lesions [130]. ID1 which is one the members of ID proteins has been shown to interact with estrogen receptor beta1 (ER*β*1) in breast cancer cells [131]. Moreover, it has been shown that ID1 expression is tightly regulated by E2 via ER genomic pathway in mouse uterus [132].

ID3 was shown to regulate mitochondrial function and morphology associated with changes in the expression of electron transport chain (ETC) complex components: CI (subunit NDUFB8), CII (subunit SDHB), CIII (subunit UQCRC2), CV (subunit ATP5A), and CIV (subunit MTCO1) [65]. Furthermore, inhibition of ID3 significantly decreased mRNA levels of mitochondrial transcription factor A (Tfam). Metabolic disorders and obesity are closely linked to higher lipid accumulation and lipogenesis. The synthesis of triglycerides (TG) is a critical step in lipogenesis process, and mitochondria facilitate the synthesis of key intermediates like glycerol 3-phosphate through the glyceroneogenic pathway and mitochondrial anaplerosis to sustain TG synthesis in the adipocyte [133]. Also, mtDNA content which is a marker for the mitochondrial number was shown to be increased significantly in white adipocytes during lipogenesis [134]. Both WAT and BAT harbor a substantial number of mitochondria [135]. Dysfunctional mitochondrial function results in increased ROS production in adipocytes and eventually results in lipid accumulation and insulin resistance [136–139]. Since ID3 is a known transcription regulator activated by PCB153, we propose that its effects on fat cell mitochondrial function may include a yet to be discovered shift in the metabolic program of adipocyte cell mitochondria. The study of the ID3 mediated mitochondrial programming holds potential promise in the prevention and treatment of obesity and metabolic disorders.

Epigenetic imprinting of adipocyte progenitor or fat stem cells by ID3 in the maternal programming of obesity shown in offspring exposed to EEDs is another plausible mechanism based on the following evidence. Specifically, we postulate that EEDs can induce ROS-mediated ID3 phosphorylation and acetylation, histone acetylation, and DNA base oxidation collectively that control expression of ID3 target genes involved in obesity and metabolic programming. This in turn controls the fate and epigenetic footprints of adipocyte progenitor cells. Reactive oxygen species like H2O2 are highly diffusible molecules. In addition to affecting ID3 signaling pathways, ROS can also facilitate histone acetylation and oxidize nuclear DNA resulting in chromatin modification. These modifications are significant because transcription in eukaryotes occurs in the context of DNA, packaged into chromatin. The basic unit of chromatin is the nucleosome, in which DNA is wrapped around the core histones H2A, H2B, H3, and H4. Acetylation of lysine in the histone tails can facilitate the opening of repressive chromatin structures in promoter regions to provide access for the transcription regulator ID3. In support of our concept that both histone acetylation and ROS-mediated DNA oxidation control the transcription of EED-induced genes, we and others have shown that E2 and PCB153-induced ROS in the nucleus, particularly H2O2, modify the surrounding DNA [49, 50, 140]. It has been recently shown that DNA oxidation through recruiting 8-oxoguanine DNA glycosylase triggers chromatin and DNA conformational changes that are essential for estrogen-mediated transcription of genes [49]. ROS generating agents and inflammation have been shown to modulate chromatin-bound hSirT1 deacetylase activity on the promoters of several genes [141–144]. Taken together, EEDs through induction of ROS may increase histone acetylations by posttranslational activation of acetylases and oxidation of DNA bases, which are necessary for ID3-mediated transcription regulation of target genes involved in obesity and metabolic complications.

Given the significant role of ID3 in stemness, it is possible that EEDs exposure may contribute to an increase in adipocyte progenitor cells. Epigenetic changes mediated by ID3 on the stem cells may ultimately increase the total number of fat cells that can be produced by an individual. Subsequent environmental exposures of these susceptible individuals who have a high number of fat stem cells, to begin with, will tend to accumulate more adipose tissue.

## 8. Interaction of ID3 and EEDs

In order to investigate how environmental exposures affect human health at genetic and protein level, we used Comparative Toxicogenomics Database (CTD) which is a publicly available database. CTD is a public website and research tool that consists of scientific data illustrating chemical-gene interactions and chemical-disease associations. CTD includes* in vitro* and* in vivo* data studies describing relationships between chemicals, genes, and diseases. The database manually curates information about EEDs-gene/protein interactions and EEDs-disease and gene-disease associations. We used these public databases to investigate the role of ID3, especially in various metabolic pathways. This tool can be used to decipher gene-environment or gene-EEDs interactions involved in the generation of metabolic diseases.

**Figure 4:**
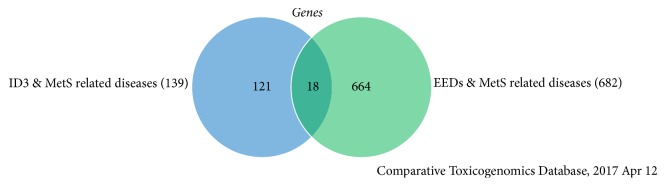
Representation of overlapping genes. Left circle summarizes ID3 and MetS-interacting genes (139), the right circle summarizes EEDs and MetS-interacting disease genes (682), and the middle area signifies overlapping genes (18) between the two groups.

**Figure 5:**
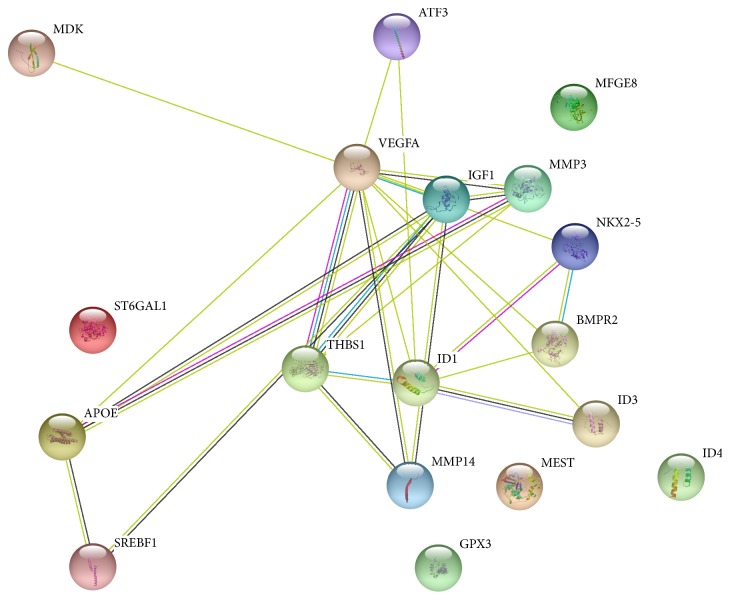
STRING protein illustration of common 18 genes to ID3 and MetS-related diseases and EEDs and MetS-related diseases.

**Figure 6:**
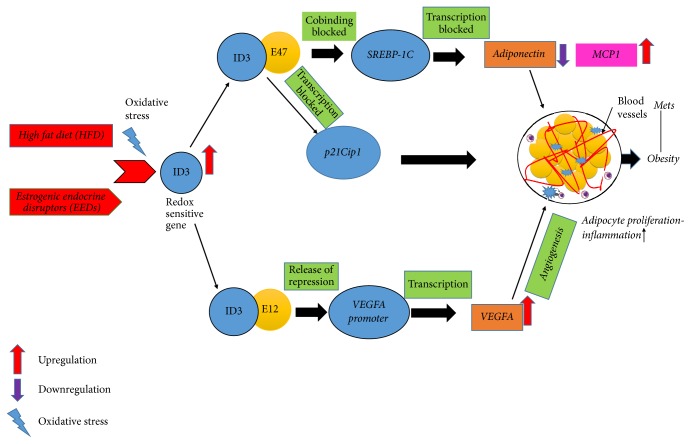
Summarization of ID3 interaction with EEDs, which may contribute to obesity.

We initially selected specific 44 EEDs as shown in Table 1 from the list of chemicals. We then chose obesity, heart block, diabetic cardiomyopathies, idiopathic pulmonary fibrosis, hyperemia, mitochondrial complex i deficiency, aortic aneurysm, metabolic syndrome X, diabetic angiopathies, cardiomyopathy, hypertension, coronary diseases, weight gain, body weight changes, overweight, and diabetes to represent MetS. Furthermore, we checked MetS-related diseases to these 44 EEDs. We established that 664 genes are overlapping in both the MetS and EEDs list of genes. Because ID3 is our candidate gene, we created a list of genes related to ID3 and MetS-related diseases. We established that 139 genes are associated with this group. Furthermore, we found 18 common genes related to both these groups demonstrated in Figure 4 and Table 2 [145]. To show an interaction between these 18 common genes, we additionally inputted them into STRING, a database of recognized and predicted protein-protein interactions. The interactions contain direct (physical) and indirect (functional) associations which stem from computational prediction, interactions aggregated from other (primary) databases, and from knowledge transfer between organisms. As seen in Figure 5 and Table 2, STRING provides a network of these 18 common proteins and furthermore provides a pathway description for the mutually represented proteins [146]. We created Table 3 with the help of STRING network, and it shows that the involvement of the ID3 protein in the various metabolic pathway. The CTD database revealed that 18 common genes associated with MetS diseases and EEDs. Furthermore, out of these 18 genes, 17 are associated with ID3. Based on these findings, we suggest there may be a molecular mechanism present between ID3/MetS-interacting genes, EED/MetS-interacting genes, and ID3/EED-interacting genes. We have summarized a potential model in Figure 6 of how EEDs-induced ROS modifies redox-sensitive ID3 protein signal transduction pathways that may contribute to the adipocytes proliferation and eventually may give rise to obesity.

## 9. Conclusion

ID3 has been shown to promote obesity in experimental models of HFD-induced obesity. Studies have reported associations between obesity and exposure to EEDs: BPA, DES, and PCBs. Based on the evidence discussed in this review, elevated exposure to EEDs or unopposed increase in the body burden of estrogen may increase the expression of the transcription regulator ID3. Although we cannot rule out the contribution of ER signaling and aromatase activity in the promotion of adipogenesis by EEDs, we propose that ID3 may be an additional molecular risk factor for obesity from environmental exposure to estrogenic chemicals. Emerging evidence demonstrated that ID3 can regulate mitochondrial function and morphology associated with changes in the expression of electron transport chain complex components and TFAM. We have previously shown that ER independent mitochondrial ROS signaling contributes to the growth of cells treated with 17*β*-estradiol [50, 56, 147]. Therefore, ID3 mediated metabolic programming of mitochondria may be dysregulated by exposure to EEDs and increase susceptibility to obesity. In addition, the ID3 dependent production of adipocytokines and recruitment of macrophages in adipose tissue are suggested to play an important role in the inflammatory process to enhance susceptibility to obesity or metabolic complications. The potential for EEDs to influence susceptibility to obesity or metabolic disorders via ID3 dependent signaling have been summarized in Figure 6. To conclude, we have systematically reviewed the existing evidence to illustrate the association between ID3, EEDs, and obesity. Furthermore, we extended this understanding of how ID3 and metabolic perturbations by environmental factors such as EEDs can increase the risk of obesity. Research is warranted to better define the influence of EEDs and ID3 gene-environment interactions on obesity. A better understanding of how ID3 and EEDs affect the risk of obesity may open up new avenues for prevention and treatment of diseases that metabolic syndrome manifest.

## Figures and Tables

**Table 1:** List of estrogen endocrine disruptors (EEDs) curated through CTD (comparative Toxicogenomics Database).

Chemical name	Chemical ID	CAS RN	Interaction count	Organism count
Bisphenol A	C006780	80-05-7	5	3
Benzo(a)pyrene	D001564	50-32-8	4	2
Coumestrol	D003375	479-13-0	3	1
Genistein	D019833	446-72-0	3	1
Titanium dioxide	C009495	13463-67-7	3	2
Cadmium chloride	D019256	10108-64-2	2	2
Carbon tetrachloride	D002251	56-23-5	2	2
Dietary fats	D004041	8016-25-9	2	1
Diethylhexyl phthalate	D004051	117-81-7	2	1
Diuron	D004237	330-54-1	2	2
Flutamide	D005485	13311-84-7	2	2
3,4-Dichloroaniline	C014464	95-76-1	1	1
4,4′-Hexafluorisopropylidene diphenol	C583074		1	1
Aluminum	D000535	7429-90-5	1	0
Amitrole	D000640	61-82-5	1	1
Ammonium chloride	D000643	12125-02-9	1	1
bis(4-Hydroxyphenyl)sulfone	C543008	80-09-1	1	1
Caffeine	D002110	58-08-2	1	1
cobaltous chloride	C018021	7646-79-9	1	1
Copper sulfate	D019327	7758-98-7	1	1
Dimethoate	D004117	60-51-5	1	1
9,10-Dimethyl-1,2-benzanthracene	D015127	57-97-6	1	1
Formaldehyde	D005557	50-00-0	1	1
Glycidol	C004312	556-52-5	1	1
Lead acetate	C008261	301-04-2	1	1
Lithium chloride	D018021	7447-41-8	1	1
Methoxyacetic acid	C013598	625-45-6	1	1
Methoxychlor	D008731	72-43-5	1	1
Methylcholanthrene	D008748	56-49-5	1	1
Methylmercuric chloride	C004925	115-09-3	1	1
Methylmercury Compounds	D008767	593-74-8	1	1
Monobutyl phthalate	C028577	131-70-4	1	1
n-Butoxyethanol	C017096	111-76-2	1	1
Nickel sulfate	C029938	7786-81-4	1	1
Nicotine	D009538	54-11-5	1	1
Octyl methoxycinnamate	C118580	5466-77-3	1	1
Perfluorooctanoic acid	C023036	335-67-1	1	1
Phenol	D019800	108-95-2	1	1
Polychlorinated biphenyls	D011078	59536-65-1	1	1
Propiconazole	C045950	60207-90-1	1	1
Quercetin	D011794	117-39-5	1	1
Oxyquinoline	D015125	148-24-3	1	1
Vinclozolin	C025643	50471-44-8	1	1
Zinc	D015032	7440-66-6	1	1

**Table 2:** 

ID3, MetS related diseases & EEDs genes
APOE
ATF3
BMPR2
GPX3
ID1
ID3
ID4
IGF1
MDK
MEST
MFGE8
MMP14
MMP3
NKX2-5
SREBF1
ST6GAL1
THBS1
VEGFA

**Table 3:** Summary of pathways involved in the 18 common genes related to ID3, MetS, and EEDs.

Pathway ID	Pathway description	Matching proteins in your network (labels)
GO.0001944	Vasculature development	APOE, BMPR2, ID1, IGF1, MFGE8, MMP14, NKX2-5, THBS1, VEGFA
GO.0072358	Cardiovascular system development	APOE, BMPR2, ID1, ID3, IGF1, MFGE8, MMP14, NKX2-5, THBS1, VEGFA
GO.0072359	Circulatory system development	APOE, BMPR2, ID1, ID3, IGF1, MFGE8, MMP14, NKX2-5, THBS1, VEGFA
GO.0001568	Blood vessel development	APOE, BMPR2, ID1, MFGE8, MMP14, NKX2-5, THBS1, VEGFA
GO.0030324	Lung development	BMPR2, ID1, IGF1, MMP14, SREBF1, VEGFA
GO.0048514	Blood vessel morphogenesis	APOE, ID1, MFGE8, MMP14, NKX2-5, THBS1, VEGFA
GO.0010941	Regulation of cell death	APOE, ATF3, ID1, ID3, IGF1, MDK, MMP3, NKX2-5, THBS1, VEGFA
GO.0050678	Regulation of epithelial cell proliferation	APOE, BMPR2, ID1, IGF1, THBS1, VEGFA
GO.0048511	Rhythmic process	ID1, ID3, ID4, MMP14, SREBF1, VEGFA
GO.0030334	Regulation of cell migration	APOE, BMPR2, IGF1, MMP14, MMP3, THBS1, VEGFA
GO.0001525	Angiogenesis	ID1, MFGE8, MMP14, THBS1, VEGFA
GO.0007623	Circadian rhythm	ID1, ID3, ID4, SREBF1
GO.0006979	Response to oxidative stress	APOE, GPX3, MMP14, MMP3, THBS1
GO.0000302	Response to reactive oxygen species	APOE, GPX3, MMP3, THBS1
GO.0031325	Positive regulation of cellular metabolic process	APOE, ATF3, BMPR2, ID4, IGF1, MDK, MMP14, NKX2-5, THBS1, VEGFA
GO.0048545	Response to steroid hormone	MDK, MFGE8, MMP14, SREBF1, THBS1
GO.0031324	Negative regulation of cellular metabolic process	APOE, BMPR2, ID1, IGF1, MMP3, NKX2-5, SREBF1, THBS1, VEGFA
GO.0045540	Regulation of cholesterol biosynthetic process	APOE, SREBF1
GO.0006950	Response to stress	APOE, BMPR2, GPX3, ID3, IGF1, MMP14, MMP3, SREBF1, THBS1, VEGFA
GO.0001935	Endothelial cell proliferation	BMPR2, MMP14
GO.0008283	Cell proliferation	BMPR2, ID4, IGF1, MMP14, NKX2-5
GO.0043536	Positive regulation of blood vessel endothelial cell migration	THBS1, VEGFA
GO.0034645	Cellular macromolecule biosynthetic process	APOE, ATF3, BMPR2, ID1, ID3, ID4, IGF1, SREBF1, ST6GAL1, THBS1
GO.0051781	Positive regulation of cell division	IGF1, MDK, VEGFA
GO.0009101	Glycoprotein biosynthetic process	BMPR2, IGF1, ST6GAL1, THBS1
GO.0008361	Regulation of cell size	APOE, BMPR2, VEGFA
GO.0043534	Blood vessel endothelial cell migration	ID1, VEGFA
GO.0016477	Cell migration	ID1, MDK, MMP14, THBS1, VEGFA
GO.0032369	Negative regulation of lipid transport	APOE, THBS1
GO.0048568	Embryonic organ development	ID3, MMP14, NKX2-5, VEGFA
GO.0045765	Regulation of angiogenesis	ID1, THBS1, VEGFA
GO.0051172	Negative regulation of nitrogen compound metabolic process	APOE, BMPR2, ID1, NKX2-5, SREBF1, VEGFA
GO.0007166	Cell surface receptor signaling pathway	BMPR2, ID1, IGF1, MDK, NKX2-5, SREBF1, VEGFA
GO.0032269	Negative regulation of cellular protein metabolic process	APOE, ATF3, IGF1, THBS1, VEGFA
GO.0045937	Positive regulation of phosphate metabolic process	APOE, BMPR2, IGF1, THBS1, VEGFA
GO.0051148	Negative regulation of muscle cell differentiation	ID3, NKX2-5
GO.0030155	Regulation of cell adhesion	IGF1, MMP14, THBS1, VEGFA
